# Interoception, alexithymia, and motor congruency: Psychological drivers of body ownership in virtual reality

**DOI:** 10.1177/03010066261447828

**Published:** 2026-05-13

**Authors:** Sarune Savickaite, Mitra Gupta, Dharshini Kannan

**Affiliations:** 1 Psychology Department, 3286 University of Exeter, Exeter, UK; 2 Department of Computer Science, 3526 University of Glasgow, Glasgow, UK; 3 School of Psychology and Neuroscience, 3526 University of Glasgow, Glasgow, UK

**Keywords:** interoception, virtual reality, individual differences, multisensory perception, alexithymia, embodied cognition, 3D perception, cognition

## Abstract

Alexithymia, a personality trait marked by difficulty in identifying and describing emotions, is associated with differences in interoception – the ability to perceive and interpret internal bodily signals. Interoception plays a key role in forming a coherent sense of self and contributes to body ownership, the feeling that one's body belongs to oneself. This study explored how interoception and alexithymia influence body ownership in immersive virtual reality, particularly under conditions of motor cue congruency. Findings revealed a negative relationship between alexithymia and interoception, and a positive effect of motor synchrony on body ownership. Interoceptive accuracy (assessed via the heartbeat counting task) showed a trend-level positive association with body ownership when virtual and physical movements were aligned. Given the modest sample size (*N* = 26) and reliance on a single cardiac interoceptive measure, findings should be considered preliminary and warrant replication in larger, multi-method studies.

## Introduction

Self-awareness is shaped by the integration of both external and internal sensory signals. While proprioception supports awareness of the body's position in space, interoception refers to the perception and interpretation of internal bodily signals such as heart rate, hunger, and respiration (Price & Hooven, 2018; Schmitt & Schoen, 2022). These internal cues contribute to emotional awareness, self-regulation, and bodily awareness and are central to the formation of a coherent sense of self (Taylor et al., 2018). Interoception consists of three related but distinct components: interoceptive accuracy (the objective ability to detect bodily signals), interoceptive sensibility (subjective confidence in bodily states), and interoceptive awareness (the metacognitive insight into bodily states; Grynberg et al., 2018; Hanley et al., 2017). In the present study, we operationalise interoception specifically as cardiac interoceptive accuracy, assessed via the heartbeat counting task (HCT). We therefore limit our empirical claims to this cardiac accuracy dimension, while acknowledging that interoception is a broader, multi-axis construct.

Individual variability in interoception has been linked to several psychological traits, one of the most prominent being alexithymia – a personality construct characterised by difficulty identifying and describing emotions (Sifneos, 1973). Individuals with high levels of alexithymia exhibit difficulties with the perception and interpretation of internal states (Herbert et al., 2011), reduced emotional intelligence (Grynberg et al., 2018), and disruptions in bodily self-awareness (Georgiou et al., 2016). Neurobiological evidence supports this link, suggesting that shared networks, particularly the insular cortex and anterior cingulate cortex (ACC), underlie both interoceptive and emotional processing (Craig, 2009; Critchley et al., 2004; Goerlich, 2018). Alexithymia has also been implicated in diminished empathy and altered social cognition and is common across various clinical populations, including those with autism, depression, and substance use disorders (Kinnaird et al., 2019; Rosaria et al., 2019).

Another foundational construct in bodily self-awareness is the experience of body ownership (EBO) – the sense that one's body belongs to oneself. EBO arises from the integration of visual, tactile, proprioceptive, and interoceptive cues (Blanke, 2012; van der Hoort et al., 2017). While relatively stable in everyday experience, body ownership is malleable under experimental manipulation. Illusions such as the rubber hand illusion (RHI) and virtual hand illusion have demonstrated how temporal and spatial synchrony between visual and tactile stimuli can induce a sense of ownership over artificial or virtual limbs (Crucianelli et al., 2018; Kocur et al., 2022). These effects are further modulated by congruency in movement, or motor cue congruency, which enhances body ownership by aligning virtual feedback with the user's physical actions (Chancel et al., 2022; Odermatt et al., 2021).

Motor cue congruency refers to the degree to which a person's real-world movements are visually matched by a virtual body or avatar in timing and form. In a congruent condition, the avatar's movements mirror the participant's own movements exactly, producing aligned visual and proprioceptive feedback. In an incongruent condition, the avatar's movements deliberately differ from the participant's real actions, creating a mismatch between visual and motor information. Research in multisensory integration suggests that such congruency enhances body ownership – the subjective sense that the virtual body belongs to oneself – whereas incongruency can reduce this experience (e.g., Sanchez-Vives et al., 2010).

Importantly, interoceptive traits appear to influence susceptibility to body ownership illusions. Individuals with higher interoceptive awareness tend to experience stronger body ownership under conditions of multisensory synchrony (Wallman-Jones et al., 2021). Conversely, high levels of alexithymia have been associated with impaired multisensory integration and reduced body ownership (Kocur et al., 2022). These findings suggest that interoception may serve as a mechanism linking emotional traits and bodily self-consciousness. Throughout, we use ‘embodiment/body ownership’ to refer to the participant's subjective experience of owning or controlling the virtual body/hand, not an intrinsic property of the avatar.

Technological advances, particularly virtual reality (VR), have enabled new ways to study and manipulate body ownership in controlled, immersive environments. VR allows for precise modulation of visual, auditory, and motor cues, making it an ideal platform to explore the intersection between sensory integration and emotional processing (Bohil et al., 2011; Haley et al., 2023). Studies show that VR can enhance interoceptive processing by focusing users’ attention on bodily states and facilitating a heightened sense of presence (Argelaguet et al., 2016; Hoyet et al., 2016). Moreover, integrating interoceptive cues into VR has potential for improving well-being and mental health outcomes (Heeter et al., 2020; Price & Hooven, 2018).

The theoretical framework guiding this study is motivated cue integration (MCI) theory, which posits that self-regulation arises from the integration of internal signals (for example, interoception), external sensory inputs (such as visual and tactile cues), and top-down goals (Shalev, 2015, 2020). According to MCI, individuals dynamically interpret sensory cues based on motivational context and goal relevance. When direct internal cues are inaccessible, individuals may rely on external sensory cues, suggesting a compensatory relationship that could influence body ownership in virtual settings.

Building on MCI theory, individuals with reduced access to internal cues (such as those with high alexithymic traits) may rely more heavily on external sensory information to regulate their actions and self-perceptions (Herbert et al., 2011; Shalev, 2015, p. 202). In VR, these external cues can be precisely manipulated, offering a unique platform to study how sensory integration shapes body ownership. When motor cues in VR are congruent with the participant's own movements, the alignment of visual and proprioceptive feedback is expected to enhance body ownership (Kocur et al., 2022; Suzuki et al., 2013). Incongruent cues introduce sensory conflict, which may reduce body ownership unless individuals compensate by weighing other available cues more heavily. According to MCI, such compensation would be especially relevant for participants with lower interoceptive awareness, who might rely more on visual-motor feedback in congruent conditions and be more disrupted by incongruency. Thus, we hypothesise that (a) congruent motor cues will enhance body ownership relative to incongruent cues, (b) higher interoceptive awareness will predict stronger body ownership in congruent conditions, and (c) higher alexithymia may be associated with reduced body ownership overall, and potentially more so in incongruent conditions. Given the limited sample size and focus on limb-level ownership (rather than agency or self-location), this prediction was considered exploratory.

## Methods

### Participants

Participants aged 18 years and above were recruited via convenience sampling. Recruitment was carried out through social media platforms (Instagram, LinkedIn, and WhatsApp) and the University of Glasgow's ‘Psychology Community Hub’ on Microsoft Teams. As the study investigated alexithymic traits within the general population, no clinical diagnosis was required. However, individuals with visual impairments or known difficulties using VR technology were advised not to participate.

A total of 26 participants took part in the study. The sample comprised 17 females (65%), seven males (27%), and two non-binary individuals (8%). While some research suggests potential biological sex differences in interoceptive accuracy and awareness (Grabauskaite et al., 2017; Khalsa et al., 2018), findings remain mixed and context-dependent. Due to the relatively small sample size in the present study, including sex as a covariate would have substantially reduced statistical power and risked overfitting any of the chosen models. As such, sex was not included in the primary analyses. We acknowledge this as a limitation and recommend that future work with larger, balanced samples examine possible interactions between sex, interoceptive traits, and body ownership in VR.

Two participants reported a clinical diagnosis of autism, and three reported a diagnosis of attention-deficit/hyperactivity disorder (ADHD). These participants were retained in the analysis, as the study aimed to examine interoceptive awareness, alexithymia, and body ownership in the general population, including individuals with neurodivergent conditions. Given the small number of participants with self-reported autism or ADHD, no separate subgroup analyses were conducted. However, this would be an interesting avenue for future research enquiries.

Participants ranged in age from 19 to 34 years (*M* = 24.8). The sample was culturally diverse, with the largest subgroup being participants from India (*n* = 8; 31%). The majority (58%) had prior experience using VR headsets for recreational or educational purposes. All participants were right-handed and used their dominant hand to perform the motor congruency task. No monetary or material incentives were offered for participation.

### Design

This study employed a repeated-measures experimental design with one within-subjects independent variable, motor cue congruency (two levels: congruent and incongruent), and two individual difference trait-level effects (‘predictors’): interoceptive awareness and alexithymia. The dependent variable was body ownership assessed after each motor congruency condition. The design allowed us to examine how motor cue congruency and individual differences in interoception, and alexithymia interact to predict body ownership in VR.

Participants completed a series of tasks: a self-report alexithymia measure, an HCT assessing interoceptive awareness, and a motor congruency task within VR. The study aimed to determine whether individual differences in interoception and alexithymia predicted changes in the subjective experience of body ownership under congruent and incongruent motor conditions. The VR-based motor congruency task was adapted from prior work developed at the Cognitive Developmental Robotics Lab, International Research Centre for Neurointelligence, University of Tokyo.

### Materials

#### Cardiac Interoceptive Accuracy (Heartbeat Counting Task)

One of the well-known tools for assessing interoception is through the HCT (Dale & Anderson, 1978). The HCT is meant to be a combination of self-report and objective measure to assess cardiac interoceptive accuracy, particularly among the cardiac axis, which is easily accessible and cost-effective to measure. The HCT combines self-report measures with an objective heart rate monitoring system in order to assess the participant's interoceptive awareness. Participants were asked to count their felt heartbeats for the span of 15 s, 30 s and 45 s and report to the experimenter. The beginning and end of the intervals were indicated by the researcher and timed. The task was scored by dividing the difference between the recorded heartbeats and the counted heartbeat recorded by a biosignal acquisition system. The HCT task was completed using the ‘BITalino heart bit’ kit, which is a low-cost modular biosignal acquisition system that allowed for measurement of cardiac activity through electrocardiography. The kit included three electrodes, a microcontroller, and a Bluetooth module for wireless data transmission (PLUX Wireless Biosignals, 2020).

#### Toronto Alexithymia Scale

Traditionally, the Toronto Alexithymia Scale (TAS-20) (Leising et al., 2009) has been used to detect and assess alexithymia among adults. The TAS-20 is a self-report scale comprised of 20 questions where participants can report subjective experience of facing difficulties in identifying and understanding feelings and thinking. The TAS-20 looked at the three non-overlapping subscales: difficulty identifying feelings, difficulty describing feeling, and external oriented thinking. The scoring for the scale is the sum of the responses for the 20 questions, with five questions being scored negatively. A score of less than 51 implies no alexithymia traits, 52–60 refers to possible alexithymia and 61 and above refers to alexithymia (Leising et al., 2009). The questionnaire has good internal consistency (Cronbach's alpha = 0.81) and was found to be stable and replicable among clinical and general population. The scale is the most widely and frequently used measure for assessing alexithymia and has been translated into 18 different languages (Taylor et al., 2003). Additionally, the full scale and the first two factors show adequate to good internal reliability in most of the translations (Leising et al., 2009; Taylor et al., 2003).

#### Body Ownership and Motor Cue Congruency Manipulation

In this study, *body ownership* refers specifically to the feeling that the virtual hand belonged to the participant, as measured by the body ownership questionnaire. While body ownership can encompass additional components such as agency and self-location, our focus here is on the *body ownership* dimension. The study on motor cue congruency was conducted in an immersive virtual environment using the Meta Quest 2 VR headset (Meta Platforms, 2024). Meta Quest 2 features an LCD display with a resolution of 1832 × 1920 pixels per eye, powered by a Qualcomm Snapdragon XR2 processor and 6 GB of RAM. It supports refresh rates of 60, 72, and 90 Hz and utilises six degrees of freedom inside-out tracking with four built-in cameras. The headset is equipped with third-generation Oculus Touch controllers and is compatible with glasses.

The virtual environment and data acquisition were managed using the Alienware m18 R1 laptop (Dell Inc., 2024). This laptop is equipped with an Intel Core i9-13980HX processor, an Nvidia RTX 4090 GPU, and 32 GB of RAM. It features an 18-inch display with a resolution of 2560 × 1600 pixels and includes 1TB of SSD storage. The operating system used was Windows 11. This high-performance setup ensured a seamless and immersive VR experience for the study.

**Figure 1.**
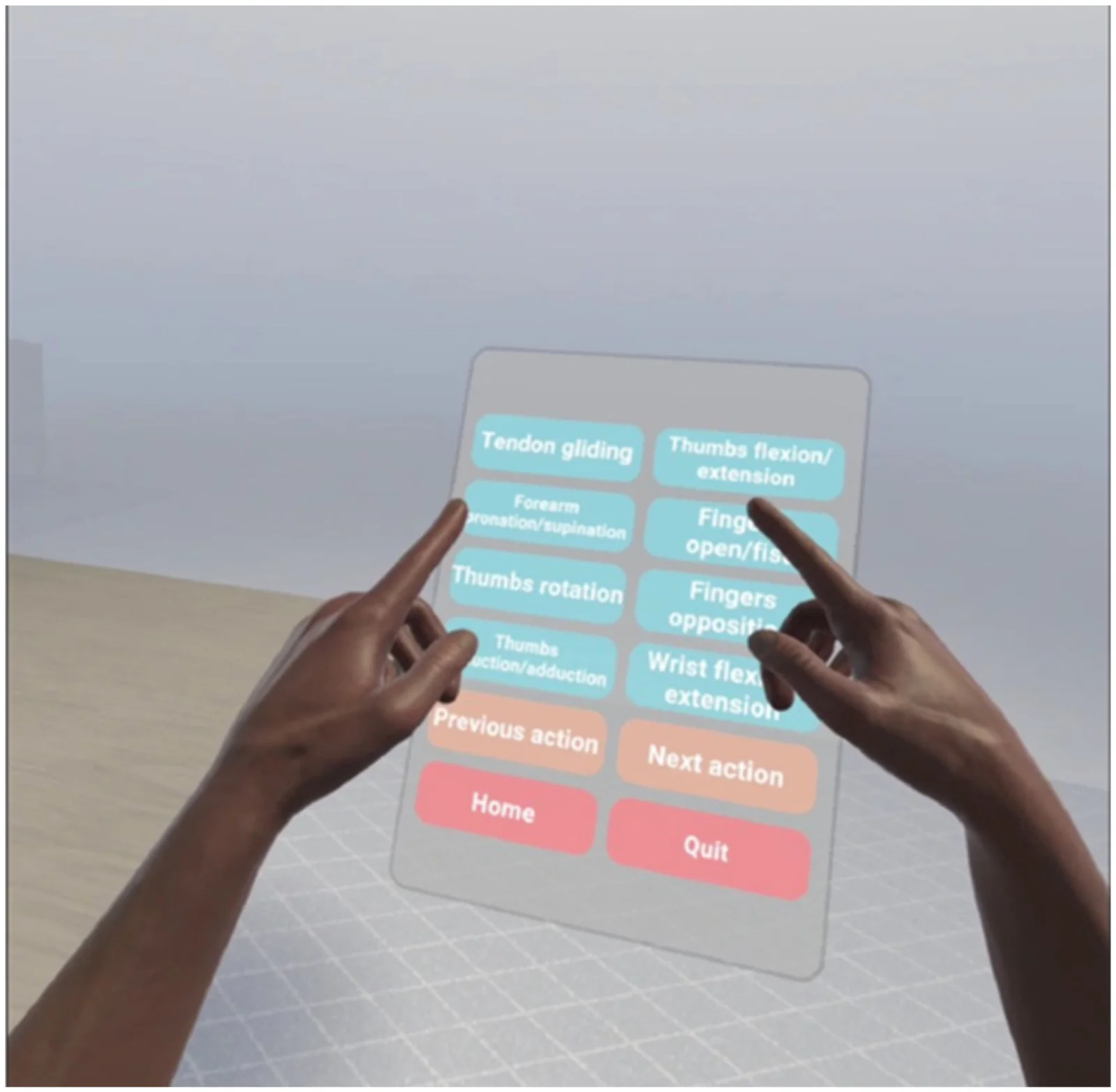
VRMT (simple movement) rehabilitation exercise options (VRMT, 2024).

A virtual mirror therapy software, ‘Virtual Reality Mirror Therapy (Simple Movement)’ (VRMT, 2024), developed by the Wearable Technology and Mobile Healthcare Lab, NCKU, Taiwan, presented a first-person virtual hand for repeated motor actions; participants’ sense of body ownership over the virtual hand was measured after each condition. The environment ‘Virtual Reality Mirror Therapy (Simple Movement)’ is an open-access software used for practising rehabilitation exercises in a virtual environment (Figure 1).

Motor cue congruency was a within-subjects independent variable with two levels (congruent, incongruent). The dependent variable was body ownership, measured using the Body Ownership Questionnaire (Drogemuller et al., 2019) immediately after each condition. The environment did not require participants to use controllers. Hand movements were played to the participants in VR, and they had to either mimic those movements (congruent condition) or produce opposite movements (incongruent condition) in real life. Within the software, multiple hand movement exercises were available (pinching motion was selected for this study), and all tasks were performed while seated.

**Figure 2.**
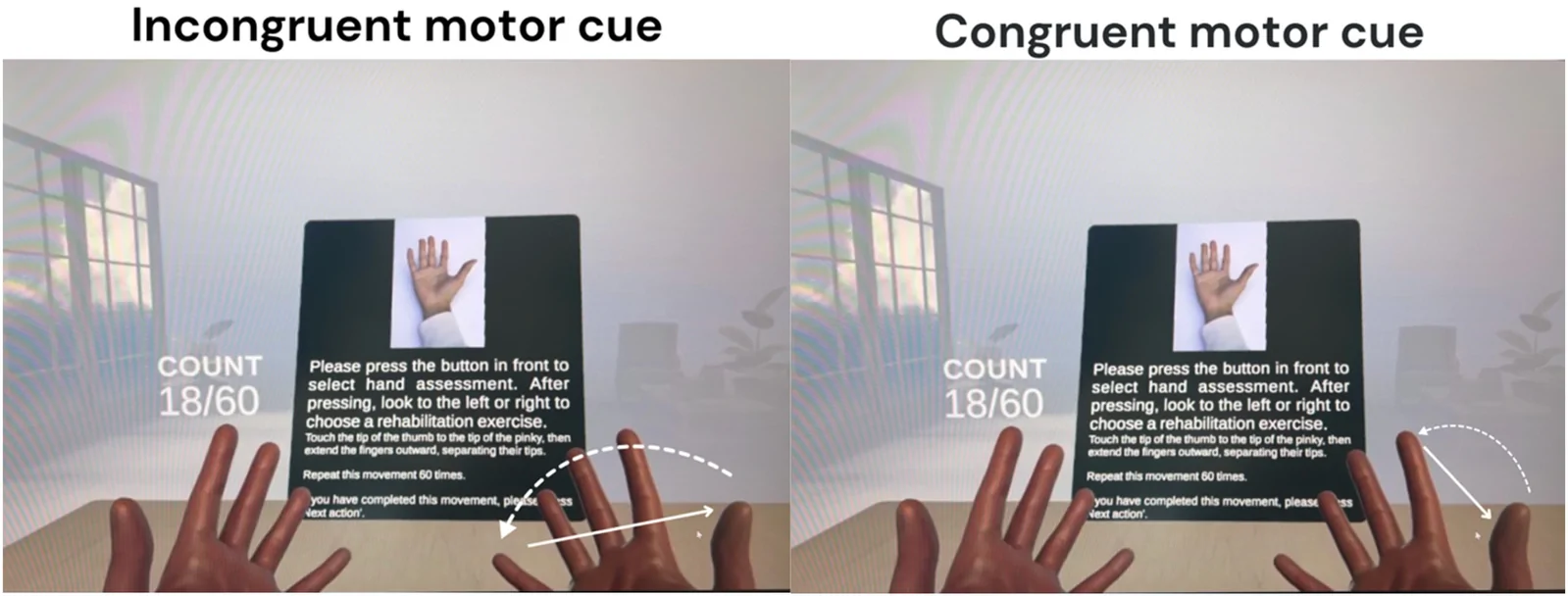
An illustration of the incongruent (left) and congruent (right) motor conditions of the virtual hand task performed from a first-person perspective within VR.

*Note.* VR = virtual reality.

The congruent condition involved participants performing the pinching motion, which was mirrored exactly by the avatar's hand. In the incongruent condition, participants still performed the pinching motion, but the avatar's hand executed the opposition movement instead, creating a mismatch between the visual and proprioceptive feedback. This ensured that motor cue congruency was manipulated purely through the alignment and misalignment of visual and motor information, with participants' actual movement held constant across conditions. Illustrations of the experiment in a virtual environment from a first-person point of view are provided in Figure 2.

The two motor conditions comprised of 30 repetitions per condition, which were repeated twice, resulting in four total motion tasks. After each repetition, body ownership was assessed through an adapted version of the arm ownership and body ownership questionnaire (Drogemuller et al., 2019), where individuals self-report their experience of body ownership in VR environment (Table 1).

**Table 1.** Arm ownership and body ownership questionnaire.

ID	Question
AO1	I felt as if the virtual third arm was my own arm
AO2	It felt as if the virtual third arm was not a part of my body
AO3	It felt as if the virtual third arm was synchronised with my motions
AO4	It felt like I could control the virtual third arm as if it was my own body part
AO5	The movements of the virtual third arm were caused by my movement
AO6	I felt as if the virtual third arm was moving by itself

The questions were adapted from Drogemuller et al. (2019) to suit the feeling of body ownership in VR. It consists of six questions that seek to answer what individuals feel during the experiment. Body ownership was quantified through gaining an average of the scores for each participant for congruent motions and incongruent motions, respectively. In the present dataset, only aggregated condition-level scores were retained for analysis. As a result, internal consistency (e.g., Cronbach's alpha) could not be recalculated for the adapted scale in this sample. The original instrument demonstrated acceptable internal reliability in prior validation studies (Drogemuller et al., 2019). However, the inclusion of ‘third hand’ items in a two-hand VR context may have introduced measurement noise and attenuated associations with trait variables.

The body ownership questionnaire included items referring to the sensation of having a ‘third hand’, which originates from supernumerary limb illusion paradigms. These items were retained to maintain comparability with previous VR body ownership studies using the same measure. However, in the present study, participants only saw a pair of virtual hands corresponding to their own. While the ‘third hand’ items were not directly applicable to this visual setup, they were included for completeness and to allow use of the validated full scale.

### Procedure

Individuals who showed interest in taking part in the study were provided with a digital consent form, which included demographic questions such as age, gender, and country of origin alongside an information sheet. Prior to participation, interested candidates were provided with an information sheet providing details on the purpose of the study, demographic focus, participants’ rights, confidentiality and contact information for further enquiries. Participants were also informed on their right to withdraw at any time by verbally reporting to the researcher during the experiment or post-participation. Digital consent was obtained after the participant showed sustained interest in participating in the study after being briefed on the experimental procedure. Additional consent was obtained to use pictures and videos recorded during the experiment for scientific dissemination purposes. Ethical approval to conduct the study was obtained from the ethics committee of the University of Glasgow.

On the day of the experiment, participants were brought into the experimental space and were briefed upon the purpose and objective of the study. Additionally, the participants were cautioned on the potential side effects of being present in a virtual environment and were asked to confirm their interest in participating in the study.

Participants were provided with the TAS-20 to fill out digitally. Participants were asked to remain seated and then briefed on the details of the HCT and asked to attach the single-use electrodes to their left ribcage based on the Einthoven triable configuration and asked to perform the task thrice for varying time intervals (15 s, 30 s and 45 s, see HCT [in Materials] for more details).

After each interval, participants were asked to report their final count of heartbeats (Desmedt et al., 2018). Finally, the main task of the study was to move the hands of an avatar in the virtual environment, followed by the questions on body ownership, where participants subjectively evaluated their experience embodying the virtual arms. The order of congruent and incongruent blocks was counterbalanced across participants. On average, the study took about 40 min per participant.

### Data Analysis

Data analysis was conducted through the aid of R (R Core Team, 2023) and R Studio (Posit Team, 2024) with packages car (Fox & Weisberg, 2019), correlation (Makowski et al., 2020), dplyr (Wickham et al., 2023), ggplot2 (Wickham, 2016), papaja (Aust & Barth, 2023), and tidyverse (Wickham et al., 2019), along with MATLAB (The MathWorks Inc., 2023).

A priori power analysis was conducted with the desired power of 0.80 to find the ideal sample required to conduct the study and concluded that the study required 36 participants as the intended sample.

#### Scoring of Interoceptive Awareness, Alexithymia, and Body Ownership

Cardiac interoceptive accuracy was quantified by using the HCT (Desmedt et al., 2018). Reported heartbeats were recorded through self-report after each time interval of the HCT task. Actual heartbeats were recorded through the ‘BITalino heart bit’ kit. Actual heartbeats were then calculated by counting the number of peaks within the generated graph for each time interval per participant through MATLAB. The final score of interoceptive awareness was calculated for each participant through the standard formula. The resulting scores for interoceptive awareness were between 0 and 1, with 0 being no interoceptive awareness and 1 being complete interoceptive awareness.

$$\begin{array}{r} \mathrm{Interoceptive}\;\mathrm{awareness}\;=\;1/3\;\Sigma \;\left(1-\left(\left|actual\;heartbeats\;-\;reported\;heartbeats\right|\right)/actual\;heartbeats\right) \end{array}$$

Similarly, alexithymia was quantified using the TAS-20, which was scored on a 5-point Likert scale, with 1 = *completely disagree* and 5 = *completely agree*. The scale consisted of 20 items, where five items were scored negatively (Items 4, 5, 10, 18 and 19), and the final score for each participant was attained by calculating the sum of the resulting scores on the 20 items. The TAS-20 used cut-off scoring where scores less than 50 resulted in non-alexithymia, 52–60 as possible alexithymia and 61 and above as alexithymia (Leising et al., 2009).

Finally, motor cue congruency was manipulated as a within-subjects factor with two levels (congruent, incongruent). Body ownership scores were obtained for each condition and averaged for analysis.

#### Statistical Analysis

Descriptive statistics were generated to understand the central tendencies and variability found within this sample for the variables interoception, alexithymia, and body ownership. Visualisation for the variable body ownership was also generated to highlight any potential outliers or anomalies.

To understand the sense of body ownership during the motion congruency task, a paired *t*-test was conducted to understand the relationship between congruent motor conditions and incongruent motor conditions within the sample. Pearson correlation coefficients with a significance threshold set as 0.05 were computed to examine the relationship between interoceptive awareness, alexithymia, body ownership, and motor cue congruency to understand the strength and direction of associations. Bonferroni correction was also carried out to adjust significance levels due to multiple comparisons (Leising et al., 2009).

Multiple linear regression analyses were conducted to assess the predictive relationship between the independent variables and the dependent variables. Regression model included interoceptive awareness, alexithymia, and motor cue congruency as trait-level effects of body ownership (Odermatt et al., 2021). Assumptions required for regression validity, including linearity, normality, and homoscedasticity, were tested, and data were transformed where necessary.

For all correlational analyses, *p*-values were reported both uncorrected and after applying Bonferroni–Holm correction to control the familywise error rate. Given the exploratory nature and small sample size of this study, uncorrected results are also reported for transparency, consistent with recommendations that caution against automatic use of overly conservative corrections (Armstrong, 2014).

## Results

The paired *t*-test comparing congruent and incongruent conditions was pre-specified and treated as confirmatory. All trait-based correlations and regression analyses were considered exploratory given the modest sample size and multiple trait-level effects.

The descriptive statistics for the key variables in a virtual environment were generated to provide an overview of the central tendencies and variability within the data set. Descriptive statistics for all participants, including age, gender, and clinical diagnosis (self-reported), are reported in Table 2 and key outcome scores in Table 3. Outlier inspection was conducted for all variables via boxplots and *z*-scores; no values exceeded ±3 *SD* from the mean, and therefore all participants were retained in analyses. The inclusion of participants with ADHD and ASD reflects the study's aim to capture a diverse sample from the general population, though this heterogeneity should be considered when interpreting the results.

**Table 2.** Participant demographics and group characteristics.

Participant	Age	Gender	Country	VR	ND
1	25	Non-binary	United States	Yes	AuADHD
2	26	Woman	India	Yes	NT
3	27	Woman	Mauritius	No	NT
4	23	Woman	United States	Yes	NT
5	28	Woman	Saudi Arabia	No	NT
6	34	Man	England	Yes	NT
7	22	Woman	India	No	NT
8	25	Man	India	No	NT
9	25	Man	Iran	Yes	NT
10	24	Woman	Iran	Yes	NT
11	23	Woman	China	Yes	NT
12	26	Man	India	No	NT
13	27	Woman	Kenya	No	NT
14	25	Woman	China	No	NT
15	24	Woman	China	Yes	NT
16	24	Woman	China	Yes	NT
17	26	Woman	Iran	Yes	NT
18	25	Man	Iran	No	NT
19	28	Non-binary	United States	No	AuADHD
20	23	Man	India	No	NT
21	27	Woman	India	Yes	NT
22	20	Woman	United States	Yes	NT
23	21	Woman	United States	Yes	NT
24	24	Woman	India	Yes	NT
25	24	Woman	India	No	NT
26	19	Man	United States	Yes	ADHD
Summary	*M* = 24.81			Yes = 15; No = 11	ND = 3, NT = 23

*Note.* VR = virtual reality experience (yes/no); ND = neurodivergent status; AuADHD = autism and ADHD; ADHD = attention-deficit/hyperactivity disorder; NT = neurotypical.

**Table 3.** Descriptive statistics for key variables.

Variable	Mean	Median	*SD*	Min	Max
Congruent motor cues	19.92	19.75	2.14	15.50	25.00
Incongruent motor cues	17.65	18.25	2.72	10.50	23.00
Interoceptive awareness	0.23	0.24	0.17	0.08	0.54
Alexithymia	52.00	51.00	8.73	38.00	70.00

*Note*. Motor cues refer to the body ownership scores.

The mean score for Congruent Motor Cues was 19.92 (*SD* = 2.14), with a median of 19.75, a minimum score of 15.50, and a maximum score of 25.00. For Incongruent Motor Cues, the mean was 17.65 (*SD* = 2.72), the median was 18.25, the minimum was 10.50, and the maximum was 23.00. The difference suggests that participants generally performed better when motor cues were congruent, as compared to incongruent motions. The larger standard deviation for incongruent motor cues implies a greater variability of performance among participants for this condition. Thus, participants of this study performed more consistently during the congruent condition.

Interoceptive awareness had a mean of 0.23 (*SD* = 0.17), a median of 0.24, a minimum of 0.08, and a maximum of 0.54. Interoceptive awareness scores were relatively low and had a symmetrical distribution. However, there was a significant individual difference in interoceptive awareness among participants, with some individuals showing very low awareness (0.23) while others lie within moderate levels of interoceptive awareness.

Lastly, alexithymia had a mean score of 52.00 (*SD* = 8.73), with a median of 51.00, a minimum score of 38.00, and a maximum score of 70.00, which implied a normal distribution of scores. There was once again a wide spectrum of alexithymia traits among this participant pool. However, the mean score for this sample falls within the ‘possible alexithymia’ (52) threshold, which indicated that the participants did not exhibit high levels of alexithymia within this sample. However, the maximum score implied that some individuals within the sample did indeed have clinically significant levels of alexithymia. Importantly, relatively few participants scored above the clinical alexithymia cut-off, suggesting restricted trait variance in this sample. This limited range may have reduced statistical power to detect associations between alexithymia and body ownership.

**Figure 3.**
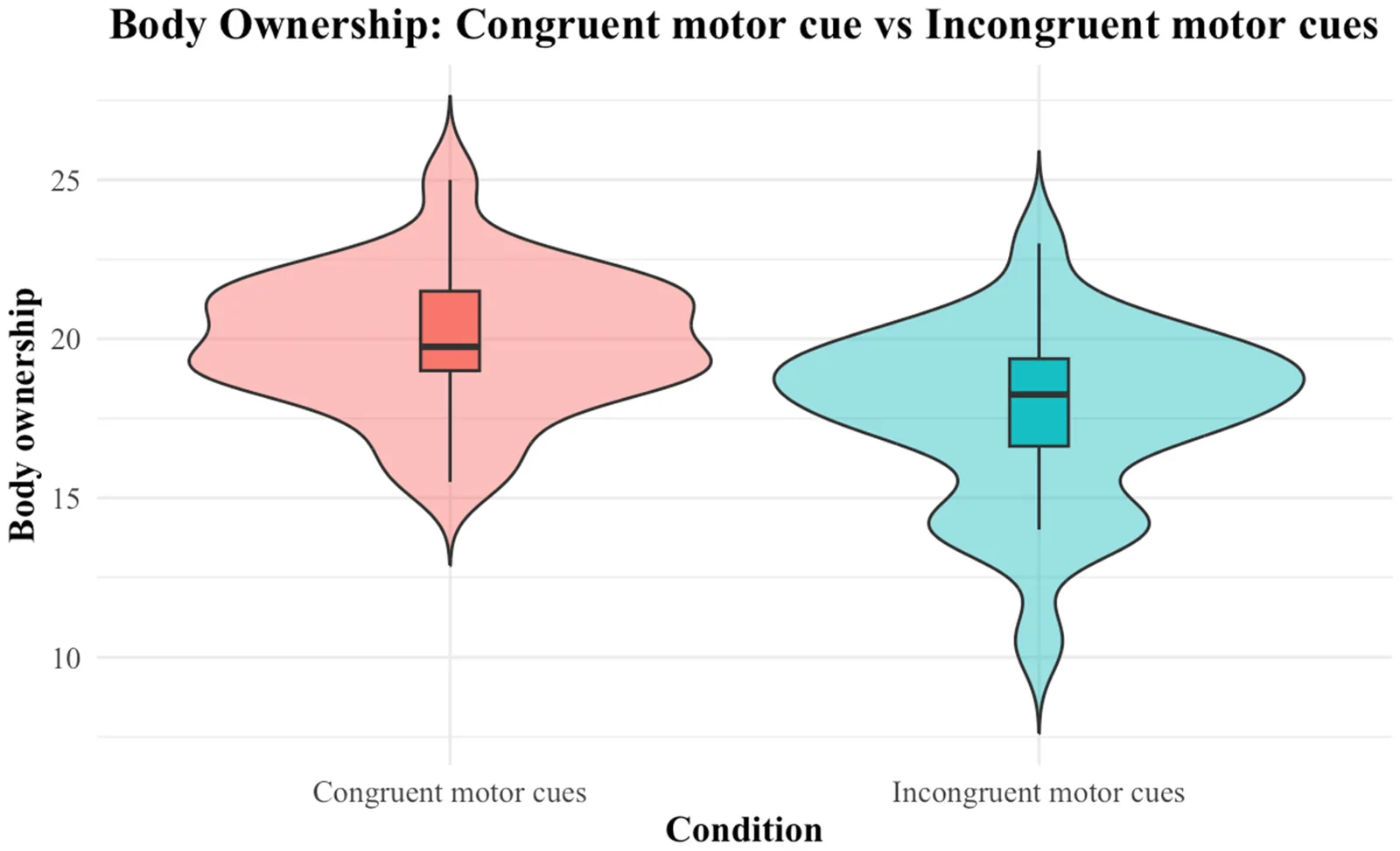
Violin boxplot comparison of body ownership in congruent motor conditions and incongruent motor cues.

A paired sample *t*-test was conducted to compare the means of incongruent motor cues to congruent motor cues to see the strength of body ownership in a virtual environment. There was a statistically significant difference in body ownership scores between the congruent and incongruent conditions, *t*(25) = 6.31, *p* < .001, 95% CI [1.53, 3.01]. This implied that on average, the participants scored 2.27 points higher on congruent motor cue conditions as compared to an incongruent motor cue condition as seen in Figure 3. Thus, the paired *t*-test showed how there was a higher sense of body ownership scores in VR during congruent motor cue conditions as compared to incongruent motor cue conditions.

A Pearson correlation analysis was conducted to examine the relationship between interoceptive awareness, alexithymia, incongruent motor cue conditions, and congruent motor cue conditions in a virtual environment. The results are summarised in Table 4.

**Table 4.** Correlation matrix for study variables.

Parameter	Interoceptive Awareness	Alexithymia	Incongruent Motor Cues
Congruent motor cues	0.467*	−0.289	0.740***
Incongruent motor cues	0.203	−0.237	–
Alexithymia	−0.484*	–	–

*Note*. Uncorrected *p*-values are reported alongside Bonferroni–Holm-adjusted *p*-values. Results significant only before correction are considered exploratory.

*p < .05. ***p < .001 (Bonferroni-adjusted).

Alexithymia and interoceptive awareness were treated as individual difference variables that could predict variations in body ownership scores within the VR conditions. As stable traits, they are not expected to be altered by the VR manipulation itself. Instead, they were examined as trait-level effects of participants’ body ownership scores in the congruent and incongruent motor cue conditions. Trait measures of interoceptive awareness and alexithymia were collected prior to the VR tasks and entered into the analyses as trait-level effects/moderators, rather than as outcomes of the experimental manipulation.

The correlation between interoceptive awareness and congruent motor cue conditions was positively correlated as *r*(26) = 0.467, *p* < .05, indicating that higher interoceptive awareness led to a stronger sense of body ownership during congruent motor cue conditions in a VR environment. Additionally, there was a negative correlation between interoceptive awareness and alexithymia, *r*(26) = −0.484, *p* < .05, which implied that higher levels of interoceptive awareness were associated with lowered levels of alexithymia in a virtual environment. However, the correlations did not remain statistically significant post the application of Bonferroni–Holm's correction.

Meanwhile, no significant correlation was found between incongruent motor cue conditions and interoceptive awareness, *r*(26) = 0.203, *p* > .05, or between incongruent motor cue conditions and alexithymia, *r*(26) = −0.237, *p* > .05. Additionally, no significant correlation was found between congruent motor cue conditions and alexithymia, *r*(26) = −0.289, *p* > .05.

Finally, multiple regression analyses were conducted to examine whether alexithymia and interoceptive awareness significantly predicted body ownership scores under congruent and incongruent motor cue conditions in the virtual environment. The results of the regression analysis for congruent motor cue conditions are presented in Table 5. Multiple regression analyses were conducted to assess the unique contributions of alexithymia and interoceptive awareness to body ownership scores under congruent and incongruent motor cue conditions. This approach clarified the predictive value of each variable while accounting for shared variance.

**Table 5.** Regression results for congruent motor cues.

Trait-Level Effects (‘Predictors’)	*B*	*SE*	*t*	*p*
Intercept	19.80	3.03	6.52	<.001
Alexithymia	−0.02	0.05	−0.39	.699
Interoceptive awareness	5.27	2.59	2.04	.053

**Figure 4.**
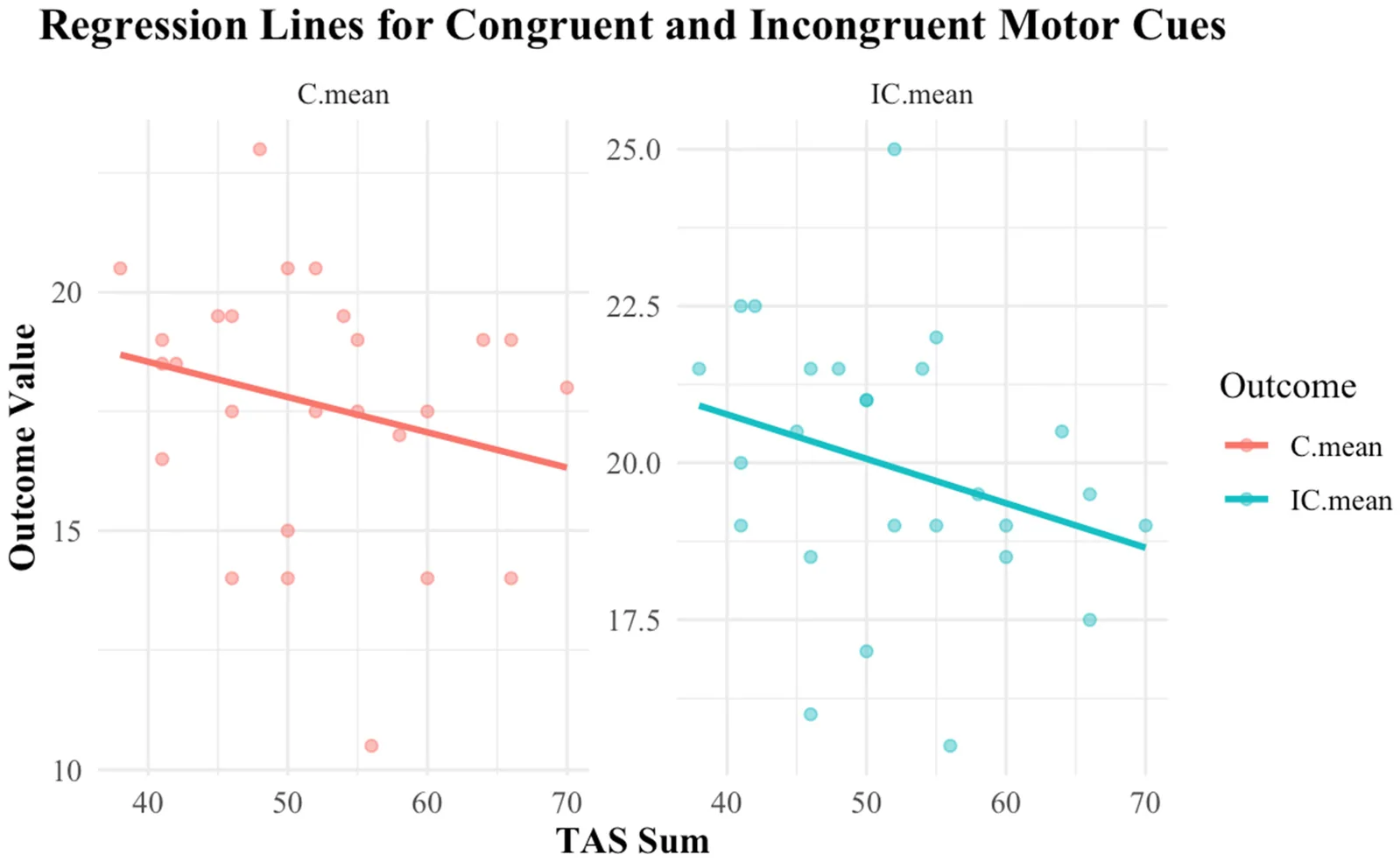
Regression plots showing the relationship between interoceptive awareness and predicted body ownership scores (outcome values) under congruent and incongruent motor cue conditions in VR (0 = *strongly disagree* to 7 = *strongly agree*). Error bars represent ±1 *SE*.

*Note.* VR = virtual reality.

Alexithymia did not significantly predict a sense of body ownership during congruent motor cue conditions, *B* = −0.02, *SE* = 0.05, *t*(25) = −0.39, *p* = .699. Yet, interoceptive awareness showed a marginally significant positive effect on body ownership during congruent motor cue conditions, with *B* = 5.27, *SE* = 2.59, *t*(25) = 2.04, *p* = .053. A regression plot has been provided below in Figure 4.

Similarly, the results of the regression analysis on incongruent motor cue conditions are presented in Table 6.

**Table 6.** Regression results for incongruent motor cues.

Trait-Level Effects (‘Predictors’)	*B*	*SE*	*t*	*p*
Intercept	20.20	4.23	4.77	<.001
Alexithymia	−0.06	0.07	−0.79	.439
Interoceptive awareness	1.81	3.61	0.50	.620

Neither alexithymia, *B* = −0.06, *SE* = 0.07, *t*(25) = −0.79, *p* = .439, nor interoceptive awareness, *B* = 1.81, *SE* = 3.61, *t*(25) = 0.50, *p* = .620, were significant trait-level effects of body ownership in incongruent motor cue conditions in a virtual environment. Thus, the regression models revealed that, within this sample, interoceptive accuracy showed a trend-level positive association of body ownership scores under the congruent motor cue condition in the virtual environment. The regression plot has been provided above in Figure 4.

## Discussion

The aim of the study was to investigate how interoceptive awareness, alexithymia, and motor cue congruency relate to body ownership scores in a virtual environment. Interoceptive awareness was measured using an HCT, alexithymia was assessed with the TAS-20 questionnaire, and body ownership was examined within an immersive motor congruency task. We expected that congruent motor cues would enhance body ownership compared to incongruent cues that higher interoceptive awareness would predict stronger body ownership scores in the congruent condition, and that higher alexithymia would be associated with reduced body ownership scores, particularly in the incongruent condition where external cues did not align with motor actions.

Contrary to our hypothesis, alexithymia was not significantly associated with body ownership in either the congruent or incongruent motor cue conditions. Several factors may explain this finding. First, the sample size was relatively small, which may have limited statistical power to detect subtle effects. Second, previous studies linking alexithymia to altered body ownership have often used paradigms involving purely visual–tactile integration (e.g., the RHI), whereas our task involved active motor control in VR, which may engage different sensory integration processes less affected by alexithymia. Finally, it is possible that alexithymia does not strongly influence motor-driven body ownership in immersive VR, suggesting that its effects may be context-specific.

The greater standard deviation observed in the incongruent motor cue condition suggests that participants’ body ownership responses to sensory–motor mismatches were more heterogeneous compared to the congruent condition. This variability may reflect individual differences in multisensory integration abilities, tolerance for sensory conflict, and prior VR experience. Participants with higher sensory adaptability may have maintained a sense of ownership despite incongruent cues, whereas others may have experienced a pronounced breakdown in body ownership. The heterogeneity of our sample, including neurodivergent participants with ADHD and ASD, may also have contributed to this broader range of responses, given the variability in sensory processing styles reported in these groups (Robertson & Baron-Cohen, 2017). Future research could explore whether such variability is systematically related to individual difference measures such as interoceptive accuracy, alexithymia, or sensory sensitivity.

### Motor Congruency and Body Ownership

Motor cue congruency plays an important role in enhancing the sense of body ownership in a virtual environment. The study found that participants performed better and felt a stronger sense of body ownership in congruent conditions (for example, when the virtual hand moved in synchronisation with their real hand) when compared to incongruent conditions (where the virtual hand did not move realistically with the movements of the participant's real hand). Participants reported a stronger sense of body ownership over the virtual hand when its movements were visually synchronised with their physical hand. Thus, simultaneous visual and tactile inputs can affect reaction time and task completion times in the motor task and improve motor performance as well as agency (Buetler et al., 2022; Yizhar et al., 2021). The findings of this study aligned with previous studies conducted by Odermatt et al. (2021), where they stated that congruency of information helped enhance motor performance in a highly embodied virtual environment. Additionally, the study conducted also showed how motor congruency and multisensory integration in uncertain conditions helped improve a sense of embodiment in a body ownership illusion among participants with high individual differences (Chancel et al., 2022; Suzuki et al., 2013).

The current study observed that participants were able to regulate sensory cues and engage with the immersive environment to achieve their task goals (Shalev, 2020). Several participants reported a sense of ownership over the virtual hand despite perceiving structural and textural differences in the virtual environment. This suggests they may have compensated for discrepancies in the virtual hand's characteristics through adaptive integration strategies (Argelaguet et al., 2016; Buetler et al., 2022; Hoyet et al., 2016). Participants also reported that, despite the task's simplicity, it elicited heightened emotions, and even minor sensory mismatches reduced their sense of ownership over the virtual limb. These findings are broadly consistent with MCI theory, although the present study did not directly test cue-weighting or prediction-error mechanisms. Future studies using graded cue reliability manipulations would be needed to evaluate this account more directly. Furthermore, the statistically significant difference in performance between congruent and incongruent motor cue conditions underscores the importance of temporal and spatial congruence in multisensory integration (Runswick et al., 2023; Suzuki et al., 2013).

### Interoceptive Awareness

Interoception is the multifaceted perceptual system that integrated signals originating from within the body. As explained in the introduction, interoception comprises of interoceptive awareness, interoceptive accuracy and interoceptive sensibility. These components collectively influence various physiological and psychological processes, such as homeostasis and self-awareness. The study conducted aligned with existing literature, emphasising the importance of interoceptive awareness in enhancing body ownership, particularly in an immersive virtual environment. It is important to note that the participants of this study showed a lowered and highly varied sense of interoceptive awareness with a mean score of 0.23 (*SD* = 0.17). However, further analysis revealed that interoceptive awareness did influence participants’ experiences in immersive VR. The insights gained from this study contribute to a broader understanding of individual differences in interoception, including those related to alexithymia.

#### Interoceptive Awareness and Alexithymia

Interoceptive awareness was found to negatively correlate with alexithymia, which is characterised by differences in identifying and verbalising emotions. The study primarily looked at the presence of alexithymia in a general population, and the sample for this study primarily lies within the threshold of ‘possible alexithymia’. The negative association between interoceptive awareness and alexithymia aligned with findings from previous studies (Butera et al., 2023; Luminet et al., 2021).

Although the correlations between interoceptive awareness and body ownership were only significant prior to Bonferroni–Holm correction, we report them transparently as exploratory effects. Overly stringent corrections, such as Bonferroni, can inflate Type II error rates and obscure potentially meaningful effects, particularly in small, hypothesis-driven studies (Armstrong, 2014). Our findings may be explained by the shared neural mechanisms underlying alexithymia and interoceptive awareness. The insula, responsible for the conscious perception of bodily states and the integration of interoceptive and emotional processes, has been shown to exhibit altered activity in individuals with alexithymia (Moriguchi & Komaki, 2013). Similarly, altered activity in the ACC has also been reported in this population (Ernst et al., 2014). Given that interoceptive awareness plays a critical role in modulating emotion regulation, these results highlight the importance of recognising and responding to internal bodily signals associated with different emotional states (Butera et al., 2023). Furthermore, the present findings align with those of Herbert et al. (2011), who reported a negative association between all facets of alexithymia, measured with the TAS-20, and interoceptive awareness, as measured by the Heartbeat Counting Task.

#### Interoceptive Awareness and Body Ownership

The study also showed that higher interoceptive awareness was positively correlated with higher body ownership scores under the congruent motor cue condition in the VR environment. The finding was consistent with previous research that highlighted the role of interoceptive awareness in improving spatial awareness and overall well-being in an immersive virtual environment (Quigley et al., 2021). The positive correlation implied that individuals with a heightened sense of interoceptive awareness can better integrate bodily signals with external stimuli, which positively improves their sense of owning a virtual avatar in VR. In this case, interoceptive awareness was highlighted when participants reported a sudden disconnect with the virtual arm when the incongruent motion did not fully emulate what the participants performed with their physical arm in the real world. Thus, the study showed that interoceptive awareness may act as a trait-level effect of higher body ownership scores under congruent motor cue conditions in a virtual environment (Buetler et al., 2022; Butler et al., 2021; Odermatt et al., 2021).

### Applications of Virtual Reality Platforms in Interoception Research

The findings of this study laid down significant implications for the use of VR in therapeutic and rehabilitative settings. Primarily, the study noted the high level of individual differences modulating the participant pool's experience in an immersive virtual environment. The experience of VR was highly subjective, and it was important to understand the role of attention and perception in the virtual experience of a simple motor task of moving a virtual limb. Moreover, the use of VR to enhance interoceptive awareness has wide-ranging benefits, from improving spatial awareness (Buetler et al., 2022; Hapuarachchi & Kitazaki, 2022) to improving emotional regulation (Rogowska & Tataruch, 2024; Shalev, 2020). VR can also be used to help improve athletic performance and physical fitness in a novel way (Rogowska & Tataruch, 2024) as well as be used to improve sensitivity towards vulnerable groups (Ventura et al., 2022) in individuals who exhibit higher levels of alexithymia, among other differences in interoception. Moreover, ensuring motor cue congruency in VR applications can enhance the user's sense of presence and engagement, making VR a more effective tool for various interventions.

The present findings may also have clinical implications for interventions targeting alexithymia and disorders characterised by disturbed body representation, such as eating disorders. Previous research has shown that individuals with high alexithymia and those with anorexia nervosa or bulimia often exhibit altered multisensory integration and reduced body ownership (Keizer et al., 2013; Scarpazza et al., 2015). VR-based body ownership tasks, such as the one used in this study, could offer an engaging and controllable environment in which to train multisensory integration and enhance body ownership. By manipulating sensory cues, VR could help individuals practise interpreting and integrating bodily signals in a safe and adaptive way, potentially improving interoceptive awareness and emotional processing. However, further work is needed to establish the efficacy and feasibility of such approaches, especially considering individual differences in sensory processing and the need for personalised interventions.

### Limitations and Future Directions

Several methodological constraints should be considered when interpreting these findings. First, the final sample (*N* = 26) fell below the a priori power estimate (*N* = 36), limiting sensitivity to detect trait-level effects and increasing the instability of regression coefficients. Second, interoception was operationalised solely via a cardiac HCT, which may partially reflect non-interoceptive processes such as beliefs or time estimation. Third, only aggregated body ownership scores were retained, preventing recalculation of internal consistency for the adapted questionnaire in the present sample. The inclusion of items originating from supernumerary limb paradigms (e.g., ‘third hand’) in a two-hand VR setup may have introduced additional measurement noise. Fourth, the sample displayed restricted variance in alexithymia scores, potentially attenuating trait–ownership associations. Together, these constraints suggest that trait-related findings should be interpreted as preliminary.

Despite its contributions, the study has several limitations. The sample size was relatively small, which may limit the generalisability of the findings. The smaller sample size may have also contributed to the statistical findings presented in the study, and a larger sample size may help in establishing stronger interaction effects among alexithymia and motor cue congruency. While the sample was highly diverse, with several individuals who reported their neurodivergent diagnosis, an increased sample size would provide a more comprehensive understanding of the relationships between interoception, alexithymia and body ownership. Investigation is required to understand the impact of individual differences in using VR technology and how multisensory integration can enable a better sense of body ownership among a larger sample size.

The study primarily focused on the cardiac axis for measuring interoceptive awareness, which may not capture the full complexity of interoception. Participants faced difficulties when they were asked to report their final heartbeat count, as they found it difficult to sense and interpret their heartbeat as the time intervals got longer. Additionally, the validity of the HCT has been questioned, as research conducted recently alludes to the HCT relying on non-interoceptive cues (Desmedt et al., 2018). Future research should explore other bodily axes and include larger, more diverse samples to validate and extend these findings. Interoceptive measures across other axes, such as the respiratory and gastrointestinal axes (Garfinkel et al., 2016) as well as novel interoceptive measures, such as using galvanic skin response (Odermatt et al., 2021).

The study's methodology could be enhanced by incorporating better VR environment and virtual avatars to understand the relationship between interoception differences and body ownership. A customisable virtual avatar, which could be modified to match skin texture and colour, can help establish a strong foundation for a sense of body ownership. Research could also look at extending the virtual avatar to embody the full body of the individual and see how full-body ownership illusions affect the perception of self in a virtual environment. Moreover, using motion capture may also help quantify motor congruency and sense of body ownership, especially within a virtual environment.

Some participants reported low confidence in their performance of the motor tasks. While this may reflect genuine difficulty, confidence ratings do not necessarily equate to task accuracy. This observation highlights a potential dissociation between subjective and objective performance, which could be investigated in future work by incorporating objective motor accuracy measures alongside self-reported confidence.

The body ownership questionnaire included items referring to a ‘third hand’, which were originally developed for supernumerary limb illusions. In the present study, participants only saw two virtual hands, and these items may have been confusing or irrelevant to the specific body ownership experience we aimed to induce. It is possible that such items could have attenuated reported body ownership, as they may have prompted participants to consider scenarios inconsistent with the visual input. Future research in similar VR contexts should consider adapting body ownership questionnaires to remove or reword such items, while ensuring the psychometric validity of the adapted instrument.

The inclusion of participants with neurodivergent conditions (ADHD and autism) in a relatively small sample introduces heterogeneity that may influence interoception, alexithymia, and body ownership. While retaining these participants aligns with the study's aim of inclusivity, it also invites caution when generalising findings to more homogeneous populations.

## Conclusion

In conclusion, this study highlighted the complex relationships between interoceptive awareness, alexithymia, and body ownership in virtual environments. Cardiac interoceptive accuracy showed a tentative positive association with body ownership under congruent motor conditions, particularly during congruent motor cue conditions in VR. Although alexithymia was negatively correlated with interoceptive awareness, it did not significantly predict body ownership in this context. The findings also demonstrated that participants could integrate mismatched sensory cues to complete the motor task, illustrating how internal and external stimuli are dynamically combined to support goal-directed behaviour. These results provide preliminary support for hypotheses derived from MCI theory, which emphasises the role of multisensory integration in self-regulation and body ownership.

The findings of this study underscore the potential of VR as a tool that may be used to enhance interoceptive awareness and body ownership, with implications for therapeutic and rehabilitative applications. Future research should continue to explore these relationships in more diverse populations and across different interoceptive measures. Introducing new conditions, such as a motion contagion task, could also be considered to understand a sense of body ownership in virtual environments.
